# Effectiveness of pelvic stabilization exercises on knee valgus, muscle activity, and strength in individuals with dynamic knee valgus

**DOI:** 10.1186/s12891-026-09556-9

**Published:** 2026-01-29

**Authors:** Mira Ambrus, Gabriella Wolf, Dóra Molnár, Badis Soussi, Tamás Horváth, Mónika Horváth, Zsombor Lacza

**Affiliations:** 1 Research Center for Sports Physiology, Hungarian University of Sports Science, Budapest, Hungary; 2 Faculty of Health Sciences, Semmelweis University, Budapest, Hungary

**Keywords:** Pelvic stabilization, Dynamic knee valgus, ACL, Training, Injury

## Abstract

**Background:**

Pelvic instability is often associated with angular deviations of lower limb and often causes valgus shift of the knee joint under load. In addition, non-contact ACL tears during sport activity are often caused by muscle weakness around the pelvis. Therefore, reinforcing the pelvic stabilizing muscles may counterbalance dynamic knee valgus (DKV). The aim of this research is to increase the activity of the pelvic stabilizing muscles through a specific exercise program and to investigate its effect of DKV after six-week pelvic stabilization training.

**Methods:**

Twenty-two subjects (male/female: 15/7) participated in the study. They performed pelvic stabilization program for six-weeks. Before and after the training, DKV was determined on both sides by performing ten single leg squats using a Kinect camera, the Gluteus maximus, medius and vastus medialis muscles activity and strength were measured by EMG system and wireless dynamometer.

**Results:**

DKV decreased from 3.15% to 1.03% for the left knee and from 3.89% to 1.26% for the right knee, the magnitude of change was significant (*p* < 0.001). Maximal Isometric Force increased significantly in all tested muscles, including the gluteus maximus, gluteus medius, and biceps femoris (all *p* < 0.05 or *p* < 0.001). This functional improvement was supported by neuromuscular changes, with EMG amplitudes increasing considerably in all assessed muscles (gluteus maximus, gluteus medius, and vastus medialis obliquus), with all six sides showing significant change (all *p* < 0.001).

**Conclusions:**

Strengthening the pelvic stabilization muscles induced a substantial improvement in knee valgus, and thus reduced the risk of developing cruciate ligament injuries. This study provides a direct link between an easy diagnosed predisposing factor a common sports injury, and offers a simple countermeasure in the form of specific exercises, that may be included against ACL injuries.

## Background

Pelvic instability is often associated with lower limb joint angular deviations and particularly causes valgus shift of the knee under load. Dynamic knee valgus is defined as an inward angulation of the knee joint within the frontal plane, which is typically marked by medial displacement of the knee in relation to the hip and foot [1, 2]. From a biomechanical perspective, valgus alignment results in increased loading on the lateral compartment and places additional stress on critical stabilizers, including the anterior cruciate ligament (ACL) and the medial collateral ligament (MCL) [1, 2]. Research involving motion analysis indicates that dynamic knee valgus, particularly during landing or cutting actions, frequently occurs alongside tibial external rotation, creating a coupled movement pattern that is linked to a heightened risk of ACL injuries, especially among female athletes [1]. Both cadaveric studies and in-vivo research have shown that valgus moments significantly raise ACL strain, particularly when they coincide with anterior tibial shear forces. Clinically, excessive valgus is correlated with altered hip mechanics, weakened hip abductors, and inadequate neuromuscular control, all of which contribute to an increased collapse in the frontal plane during weight-bearing activities [1, 2]. The mentioned phenomenon is often observed as musculoskeletal disorder in different sports and daily life. As the knee stabilizer muscles originate from the pelvis, there is considerable interaction between the knee joint, the pelvis and the hip joint [3, 4]. In addition, non-contact anterior cruciate ligament (ACL) ruptures during sports activities are often caused by pelvic muscle weakness and dynamic valgus position [5]. ACL injuries are widespread in the world. In the United States each year 1 out of 3500 people suffer from ACL injury. Studies showed that the most common reasons for dynamic knee valgus are the lack of appropriate gluteus strength [4, 6], weak quadriceps [7] or weak hamstring muscles [8]. Several studies showed that weakness of m. gluteus medius and m. gluteus maximus can lead to an increased dynamic knee valgus position [9, 10]. Excessive knee valgus is a predisposing factor for osteoarthritis (OA), patellofemoral pain syndrome and ACL rupture [11–13] and may also lead to sport injuries. Several studies show that skiing, ball games and martial arts are the most dangerous types of sports regarding knee injuries [12, 14]. Around 70% of all ACL injuries come from sports participation, especially skiing, football, soccer, baseball and basketball [15]. ACL rupture alone is also known as predisposing factor for knee osteoarthritis [16]. It is worth noting that, ACL-injured athletes develop knee osteoarthritis symptoms earlier than those without ACL injuries [17]. Moreover, there is a strong relationship between the lower extremity valgus during dynamic activities and lower extremity injuries such as patellofemoral pain syndrome or anterior cruciate ligament injury [18]. In 2018 Dix et al. found a relationship between hip muscle strength and dynamic lower extremity valgus [18]. Therefore, reinforcing the physiological function of the pelvic stabilizing muscles may counterbalance dynamic knee valgus. Functional stabilization training, which include hip muscle strengthening, lower limb and trunk movement control exercises, modified the knee kinematics in the frontal plane during single leg squat by recreational female athletes with patello-femoral pain syndrome (PFPS). Functional Stabilization Training (FST), as outlined by Baldon et al. (2014) [19], integrates exercises for hip strengthening, core stabilization, and movement control, all aimed at enhancing lower-limb alignment during functional, weight-bearing activities. This methodology addresses the neuromuscular deficiencies commonly linked to Dynamic Knee Valgus (DKV), including weak hip abductors, inadequate trunk control, and excessive hip adduction or internal rotation during single-leg movements. In the study [19], women suffering from patellofemoral pain who underwent eight weeks of FST exhibited notable improvements in lower-extremity kinematics suggesting that FST is effective in rectifying improper frontal-plane mechanics and can significantly diminish the movement patterns associated with dynamic knee valgus [19]. Most studies [7] reported that exercise intervention programs can have a significant positive effect on improving dynamic knee valgus. Several other studies show that preventive training programs can decrease the incidence of non-contact lower limb, knee, ACL or ankle injuries. At the same time there is a need to determine exactly the essential elements of a successful training program [20]. There are several methods to evaluate the dynamic knee valgus in an office setting. Our study used the Kinect Azure 3D camera, which can detect joints and capture motion without markers.

This study aimed to increase the activity of the pelvic stabilizing muscles through a specific exercise program and to investigate its effect on dynamic knee valgus under load.

## Methods

###  Participants 

Twenty-two (male/female: 15/7; Age = 34.3 ± 8.9 yrs.) healthy and physically active participants were involved in the study. Inclusion criteria were age between 18 and 50 years, no history of lower limb injury, and a relative dynamic knee valgus greater than 2% of lower limb length during single-leg squat. The 2% DKV was measured at 15% squat depth. Participants with recent musculoskeletal pain, neurological disorders, or conditions limiting exercise participation were excluded. The overall well-being of the subjects was evaluated by the standard SF-36 score [21], the level of sports activity was assessed by the Tegner score [22] and subjective knee function by the Lysholm score [23]. Anthropometric data and the baseline characteristics are summarized in Table 1.

**Table 1:** Participant’s anthropometric characteristics

Anthropometric parameters	value mean ± SD
Age (yrs)	34.3 ± 8.9
Sex (male/female)	15/ 7
Height (cm)	177.2 ± 7.3
Weight (kg)	75.4 ± 9.9
BMI (kg⋅m^− 2^)	24.0 ± 2.5
dominant leg (left/right)	6 / 16

All subjects provided written informed consent. The experimental procedures were approved by the ethics committee (Ethical license number: TE-KEB/22/2021).

###  Procedures 

Before starting the protocol, subjects provided informed consent, which detailed the study’s purpose, protocols, and procedures, all details were explained to each player. All participants participated in a familiarization session before the data collection phase to acquire knowledge of the proper technique for executing the single-leg squat and the exercises incorporated in the training regimen. Upon providing written informed consent, participants undertook baseline evaluations, which included questionnaires (SF-36, Tegner, and Lysholm scores) as well as anthropometric measurements. Subsequently, dynamic knee valgus, muscle activity, and isometric muscle strength were documented. After the baseline assessments, participants engaged in a six-week pelvic stabilization training program aimed at enhancing the strength of the gluteus maximus, gluteus medius, and vastus medialis obliquus muscles, while also improving pelvic control. Training sessions occurred three times weekly and were systematically intensified through three distinct phases. At the conclusion of the six-week intervention, all baseline measurements were repeated under the same conditions.

### Measurements

#### Dynamic knee valgus assessment

DKV was assessed by using a Microsoft Azure Kinect camera system (Microsoft Corp. Redmond, WA USA) and Dynaknee software (Dynaknee, OrthoSera Kft, Budapest, Hungary) for Windows 10 operation system that allowed data management, recording and analysis [24]. Kinect Azure contains an RGB (red, green and blue) camera and a three dimensional infrared depth sensor, thus it is able to measure the full body kinematics [24]. Kinect Azure estimates 3 coordinates of every major joint of the human body in 3 planes without any marker. During the examination, the camera was setup 250 cm away from the subjects and 100 cm height from the ground, thus provided ideal circumstances to capture a full-body image. The valgus data were collected with the help of the Dynaknee software. The initial position involved standing upright with hands placed on the hips to gather baseline data regarding the length of the lower extremities. For the movement excursion, the subjects flexed their left knee to a 90-degree angle, resulting in the left lower leg being elevated and aligned parallel to the ground. To assess the DKV shift, participants executed ten well-performed single-leg squats with both legs (Dominant and non-Dominant), aiming to descend as low as possible while ensuring that their heel and foot remained in contact with the ground. This method places the knee under significant load, thereby inducing a DKV under tension. Squats were deemed valid if participants sustained their balance throughout the repetition with their hands on their hips and refrained from stepping away during the assessment. The movement was recorded and analyzed by the software and the Kinect Azure camera. Knee valgus quantification was performed at 15% of the maximum squat depth expressed in % of lower limb length [24].

#### Electromyography (EMG)

Gluteus maximus, medius and vastus medialis muscle EMG signals were acquired with a NeuroTrac Simplex 1 channel EMG device. The configuration included a laptop designated for data recording, the EMG device was connected to a portable station where bipolar surface electrodes were affixed, a designated area for skin preparation, and a space allocated for executing the movements. The skin was prepared through shaving and disinfection to minimize the impedance between the electrodes. Subsequently, the bipolar non-invasive electrodes were positioned at both the proximal and distal ends of the muscles. Additionally, an electrode was affixed to the patella to establish a baseline, ensuring it was placed on a bony surface with minimal soft tissue to prevent interference from muscle activity. The localization of the electrodes was conducted through palpation, and the procedure for the measurement adhered to SEMG guidelines. The acquisition protocol comprised 5 cycles of a 5 s maximal isometric contraction and a subsequent 5 s relaxation phases. The cycle-averaged EMG amplitudes recorded according to the manufacturer’s guidelines in µV. 

#### Maximum isometric muscle force measurements

Maximum isometric force of the gluteus maximus, gluteus medius and the biceps femoris muscles were registered with a Hoggan MicroFET 3 wireless dynamometer. Participants applied pressure for 5 s against the sensor of the dynamometer. On alternating sides, the procedure was repeated 3 times/side and the average force on each side is reported in N. Force measurements were carried out with the help of two physiotherapists, who assisted fixing the participant’s pelvis in the optimal position isolating the examined muscle during the procedure. 

###  Intervention

The six-week pelvic stabilization program was designed according to the FITT principle (Frequency, Intensity, Time, and Type) to improve neuromuscular control and movement quality rather than maximal strength. The primary objective was to enhance selective activation of deep and pelvic stabilizer muscles and to integrate this control into functional movement patterns. Two supervised sessions per week (40–45 min each) were conducted for six consecutive weeks, complemented by daily 15–20 min home-based exercises supported by instructional videos. Exercises were performed at a moderate effort level corresponding to RPE 12–14. Intensity was progressively increased by raising the number of repetitions, reducing rest intervals, gradually involving limb movements, and introducing unstable surfaces and light perturbations. Each supervised session lasted 40–45 min, including a mobility warm-up, the main stabilization segment, and 10–15 min of stretching and recovery work at the end. Exercises consisted of core and pelvic stabilization movements, such as transversus abdominis activation in supine position, gluteus maximus and gluteus medius strengthening in various postures, squats and lunges with resistance bands, static and dynamic planks, and proprioceptive balance tasks on stable and unstable surfaces (e.g., balance pads or Pilates balls). In the final phase, low amplitude jump and controlled landing drills were introduced to integrate postural control into dynamic movement patterns and to enhance functional stability. The program consists of 3 phases:

- Phase 1 (Weeks 1–2): Focused on segmental activation of deep stabilizers (transversus abdominis, multifidus) and selective gluteal activation (gluteus maximus and medius) without excessive recruitment of synergistic muscles, while maintaining physiological lumbar lordosis. Training included simple static and low-load motor control exercises performed on stable surfaces.
- Phase 2 (Weeks 3–4): Combined static and dynamic activation of the gluteal and quadriceps muscles while maintaining transversus abdominis contraction and physiological lumbar lordosis. Training included functional squats and lunges with resistance bands and proprioceptive drills performed on stable and progressively unstable surfaces. The focus was on integrating pelvic and core stability into controlled, multi-joint functional movements.
- Phase 3 (Weeks 5–6): Added dynamic perturbations, single-leg stability tasks, and controlled landing and stabilization exercises to integrate postural stability into functional movement. The focus was on maintaining lumbopelvic control and core activation during dynamic activities.

### Statistical methods

Normality of the pre- and post-data was checked using the Shapiro Wilk’s normality test. To analyze the pre-post intervention results, paired-sample *t*-test and the non-parametric Wilcoxon signed rank test were employed depending on data normality. Statistical significance was considered when *p* < 0.05. Data analysis was performed with R (version 4.1.2).

## Results

Tables 2 and 3 display the descriptive statistics and the differences for T1 (pre) and T2 (post) of the Lyshlom, Tegner and SF-36 survey questionnaires. While Tegner scores did not change from the pre (T1) and posttest (T2) evaluations indicating general activity levels, Lysholm scores describe knee pain complaints improved after the training session. Moreover, overall well-being SF-36 survey scores also improved by the second evaluation (Table 4). The statistical results for the T-test and Wilcoxon test are presented in Table 4 and the results of test of normality are presented in Table 5.

**Table 2:** Descriptive statistics for T1 and T2

			95% Confidence Interval Mean			
	Median	Mean	Lower	Upper	Std. Deviation	IQR
Lysholm_T1	90.000	89.652	85.149	94.155	10.412	12.500
Lysholm_T2	100.000	94.739	90.151	99.327	10.610	5.000
Tegner_T1	4.000	4.391	3.705	5.078	1.588	2.500
Tegner_T2	4.000	4.348	3.517	5.179	1.921	2.500
SF_36_phys_funct_T1	100.000	95.000	91.370	98.630	8.394	10.000
SF_36_phys_funct_T2	100.000	96.957	94.820	99.093	4.940	5.000
SF_36_role_lim_phys_health_T1	100.000	90.217	78.583	101.851	26.904	0.000
SF_36_role_lim_phys_health_T2	100.000	98.913	94.987	102.833	5.213	0.000
SF_36_role_lim_emotional_T1	100.000	79.704	64.189	95.220	35.880	33.350
SF_36_role_lim_emotional_T2	100.000	89.852	77.999	101.705	27.410	0.000
SF_36_energy/fatigue_T1	65.000	64.348	56.145	72.550	18.968	15.000
SF_36_energy/fatigue_T2	80.000	75.870	67.684	84.055	18.929	20.000
SF_36_emo_well-being_T1	80.000	76.870	70.993	82.746	13.589	18.000
SF_36_emo_well-being_T2	88.000	84.174	78.015	90.333	14.244	8.000
SF_36_social_funct_T1	87.500	80.978	72.374	89.582	19.897	37.500
SF_36_social_funct_T2	100.000	88.587	80.132	97.042	19.552	12.500
SF_36_pain_T1	80.000	76.522	67.850	85.193	20.053	22.500
SF_36_pain_T2	100.000	92.283	87.109	97.456	11.964	10.000
SF_36_gen_health_T1	85.000	81.739	75.398	88.080	14.664	17.500
SF_36_gen_health_T2	90.000	87.391	81.457	93.326	13.724	20.000

**Table 3:** Descriptive statistics for differences T2-T1

			95% Confidence Interval Mean			
	Median	Mean	Lower	Upper	Std. Deviation	IQR
Lysholm_diff	6.000	5.087	1.500	8.674	8.295	10.000
Tegner_diff	0.000	-0.043	-0.763	0.676	1.665	1.500
SF_36_phys_funct_diff	0.000	1.957	-0.370	4.283	5.381	5.000
SF_36_role_lim_phys_health_diff	0.000	8.696	-1.921	19.312	24.551	0.000
SF_36_role_lim_emotional_diff	0.000	10.148	-6.933	27.229	39.500	0.000
SF_36_energy/fatigue_diff	10.000	11.522	4.860	18.184	15.406	22.500
SF_36_emo_well-being_diff	8.000	7.304	0.103	14.506	16.653	12.000
SF_36_social_funct_diff	0.000	7.609	-1.573	16.791	21.233	25.000
SF_36_pain_diff	10.000	15.761	7.467	24.055	19.180	27.500
SF_36_gen_health_diff	5.000	5.652	2.505	8.799	7.278	10.000

**Table 4:** One sample T-Test for differences T2-T1

	Test	Statistic	df	*p*	Effect Size	SE Effect Size
Lysholm_diff	Student	2.941	22	.008	0.613	0.227
	Wilcoxon	128.500		0.014	0.680	0.269
Tegner_diff	Student	-0.125	22	0.901	-0.026	0.209
	Wilcoxon	69.500		0.958	0.022	0.277
SF_36_phys_funct_diff	Student	1.744	22	0.095	0.364	0.215
	Wilcoxon	59.000		0.113	0.513	0.316
SF_36_role_lim_phys_health_diff	Student	1.699	22	0.103	0.354	0.215
	Wilcoxon	6.000		0.174	1.000	0.554
SF_36_role_lim_emotional_diff	Student	1.232	22	0.231	0.257	0.212
	Wilcoxon	21.000		0.240	0.500	0.399
SF_36_energy/fatigue_diff	Student	3.587	22	**0.002**	0.748	0.236
	Wilcoxon	153.000		**0.003**	0.789	0.262
SF_36_emo_well-being_diff	Student	2.104	22	**0.047**	0.439	0.218
	Wilcoxon	131.500		**0.046**	0.538	0.262
SF_36_social_funct_diff	Student	1.719	22	0.100	0.358	0.215
	Wilcoxon	89.000		0.100	0.483	0.285
SF_36_pain_diff	Student	3.941	22	**< 0.001**	0.822	0.241
	Wilcoxon	142.500		**0.002**	0.863	0.269
SF_36_gen_health_diff	Student	3.725	22	**0.001**	0.777	0.238
	Wilcoxon	138.000		**0.003**	0.804	0.269

CI could not be computed for effect size, due to low sample size and/or extreme effect size

For the Student t-test, effect size is given by Cohen’s *d*. For the Wilcoxon test, effect size is given by the matched rank biserial correlation

For the Student t-test, the alternative hypothesis specifies that the mean is different from 0. For the Wilcoxon test, the alternative hypothesis specifies that the median is different from 0

Sample size too small for desired confidence level. Using 0.0% instead

Bold = Significance (*p* < 0.05)

**Table 5:** Test of normality (Shapiro-Wilk)

	W	*p*
Lysholm_diff	0.953	0.336
Tegner_diff	0.897	0.022
SF_36_phys_funct_diff	0.905	0.032
SF_36_role_lim_phys_health_diff	0.412	**< 0.001**
SF_36_role_lim_emotional_diff	0.753	**< 0.001**
SF_36_energy/fatigue_diff	0.969	0.655
SF_36_emo_well-being_diff	0.914	0.049
SF_36_social_funct_diff	0.917	0.057
SF_36_pain_diff	0.934	0.131
SF_36_gen_health_diff	0.948	0.261

Significant results suggest a deviation from normality

Bold = Significance (*p* < 0.05)

Measurement results of the muscles activity are displayed as median and IQR. As a outcome of training program, the EMG amplitudes were increased considerably on both sides (Table 8). We observed the least change (38 µV) in case of the right gluteus maximus, while the right vastus medialis EMG increased the most (98 µV). EMG descriptive statistics and pre-post comparison (T1-T2) results are summarized in Tables 6, 7 and 8. The check of normality is presented in Table 9.

**Table 6:** Descriptive statistics for T1 and T2

			95% Confidence Interval Mean			
	Median	Mean	Lower	Upper	Std. Deviation	IQR
GM_D_T1	121.0	190.0	116.2	263.8	170.6	149.5
GM_ND_T1	119.0	181.2	105.3	257.1	175.5	131.0
GM_D_T2	165.0	243.6	156.4	330.7	201.5	198.5
GM_ND_T2	195.0	253.7	170.1	337.3	193.3	145.5
GMED_D_T1	170.0	231.3	160.8	301.8	163.0	168.0
GMED_ND_T1	172.0	226.2	165.3	287.1	140.8	132.0
GMED_D_T2	236.0	314.9	220.1	409.7	219.2	183.0
GMED_ND_T2	234.0	318.3	218.1	418.6	231.8	187.0
VMO_D_T1	218.0	281.0	207.7	354.4	169.7	140.0
VMO_ND_T1	216.0	261.1	202.0	320.2	136.7	136.5
VMO_D_T2	316.0	362.7	290.4	435.0	167.3	173.5
VMO_ND_T2	271.0	340.4	271.6	409.2	159.1	161.5

**Table 7:** Descriptive statistics for differences T2-T1

					95% Confidence Interval Mean			
	Valid	Missing	Median	Mean	Lower	Upper	Std. Deviation	IQR
GM_D	23	0	22.00	53.57	25.07	82.06	65.90	62.50
GM_ND	23	0	65.00	72.48	39.13	105.82	77.11	84.50
GMED_D	23	0	64.00	83.57	29.34	137.79	125.39	69.00
GMED_ND	23	0	41.00	92.13	30.40	153.86	142.76	94.00
VMO_D	23	0	72.00	81.65	51.38	111.93	70.01	92.00
VMO_ND	23	0	65.00	79.26	47.53	111.00	73.39	115.00

**Table 8:** One sample T-Test for differences T2-T1

	Test	Statistic	df	*p*	Effect Size	SE Effect Size
GM_D	Student	3.898	22	< 0.001	0.813	0.241
	Wilcoxon	276.000		**< 0.001**	1.000	0.234
GM_ND	Student	4.508	22	**< 0.001**	0.940	0.250
	Wilcoxon	275.000		**< 0.001**	0.993	0.234
GMED_D	Student	3.196	22	**0.004**	0.666	0.231
	Wilcoxon	276.000		**< 0.001**	1.000	0.234
GMED_ND	Student	3.095	22	0.005	0.645	0.229
	Wilcoxon	271.000		**< 0.001**	0.964	0.234
VMO_D	Student	5.594	22	**< 0.001**	1.166	0.270
	Wilcoxon	272.000		**< 0.001**	0.971	0.234
VMO_ND	Student	5.180	22	**< 0.001**	1.080	0.262
	Wilcoxon	276.000		**< 0.001**	1.000	0.234

CI could not be computed for effect size, due to low sample size and/or extreme effect size

For the Student t-test, effect size is given by Cohen’s *d*. For the Wilcoxon test, effect size is given by the matched rank biserial correlation

For the Student t-test, the alternative hypothesis specifies that the mean is different from 0. For the Wilcoxon test, the alternative hypothesis specifies that the median is different from 0. Bold = Significance (*p* < 0.05)

**Table 9:** Test of normality (Shapiro-Wilk)

	W	*p*
GM_D	0.721	< 0.001
GM_ND	0.779	**< 0.001**
GMED_D	0.514	**< 0.001**
GMED_ND	0.609	**< 0.001**
VMO_D	0.936	0.149
VMO_ND	0.881	0.011

Significant results suggest a deviation from normality

Bold = Significance (*p* < 0.05)

Pre- and post-training contractile force of the gluteus maximus, gluteus medius and biceps femoris muscles displayed in Tables 10 and 11. As anticipated, the training program increased muscle force in all muscles (Table 12). The statistical analyses and time comparison (T1-T2) are presebted in Table 12 and the normality check in table Table 13.

**Table 10:** Descriptive statistics for T1 and T2

			95% Confidence Interval Mean			
	Median	Mean	Lower	Upper	Std. Deviation	IQR
GM_D_1	219.0	224.8	202.0	247.5	52.56	81.17
GM_ND_1	235.0	234.5	212.2	256.8	51.47	83.16
GM_D_2	270.7	260.1	229.2	291.0	71.53	97.16
GM_ND_2	270.3	259.9	230.3	289.5	68.42	99.00
BiFem_D_1	121.7	133.9	116.4	151.3	40.37	54.50
BiFem_ND_1	128.3	133.6	115.8	151.4	41.18	52.00
BiFem_D_2	159.0	163.5	145.7	181.2	41.11	44.00
BiFem_ND_2	147.3	155.3	137.4	173.2	41.33	52.33
GMED_D_1	115.7	113.6	104.7	122.6	20.71	34.17
GMED_ND_1	115.7	114.3	103.3	125.2	25.34	33.17
GMED_D_2	139.3	147.5	134.4	160.6	30.29	28.16
GMED_ND_2	153.0	146.5	135.9	157.1	24.49	34.50

**Table 11:** Descriptive Statistics for differences T2-T1

			95% Confidence Interval Mean			
	Median	Mean	Lower	Upper	Std. Deviation	IQR
GM_D_diff	28.000	35.36	11.782	58.94	54.53	48.83
GM_ND_diff	12.330	25.39	1.512	49.27	55.22	38.00
BiFem_D_diff	20.670	29.58	18.765	40.39	25.01	36.34
BiFem_ND_diff	9.330	21.70	10.126	33.27	26.76	25.00
GMED_D_diff	29.670	33.90	23.540	44.26	23.95	33.51
GMED_ND_diff	25.000	32.20	21.978	42.43	23.64	34.51

**Table 12:** One Sample T-Test for differences T2-T1

	Test	Statistic	df	*p*	Effect Size	SE Effect Size
GM_D_diff	Student	3.110	22	0.005	0.648	0.229
	Wilcoxon	248.000		**< 0.001**	0.797	0.234
GM_ND_diff	Student	2.205	22	0.038	0.460	0.219
	Wilcoxon	243.000		**0.001**	0.761	0.234
BiFem_D_diff	Student	5.672	22	**< 0.001**	1.183	0.272
	Wilcoxon	276.000		**< 0.001**	1.000	0.234
BiFem_ND_diff	Student	3.889	22	**< 0.001**	0.811	0.240
	Wilcoxon	267.000		**< 0.001**	0.935	0.234
GMED_D_diff	Student	6.787	22	**< 0.001**	1.415	0.295
	Wilcoxon	276.000		**< 0.001**	1.000	0.234
GMED_ND_diff	Student	6.532	22	**< 0.001**	1.362	0.289
	Wilcoxon	275.000		**< 0.001**	0.993	0.234

CI could not be computed for effect size, due to low sample size and/or extreme effect size

For the Student t-test, effect size is given by Cohen’s *d*. For the Wilcoxon test, effect size is given by the matched rank biserial correlation

For the Student t-test, the alternative hypothesis specifies that the mean is different from 0. For the Wilcoxon test, the alternative hypothesis specifies that the median is different from 0. Bold = Significance (*p* < 0.05)

**Table 13:** Test of Normality (Shapiro-Wilk)

	W	*p*
GM_D_diff	0.902	0.027
GM_ND_diff	0.843	**0.002**
BiFem_D_diff	0.877	0.009
BiFem_ND_diff	0.794	**< 0.001**
GMED_D_diff	0.908	0.037
GMED_ND_diff	0.944	0.222

Significant results suggest a deviation from normality

Bold = Significance (*p* < 0.05)

**Fig. 1:**
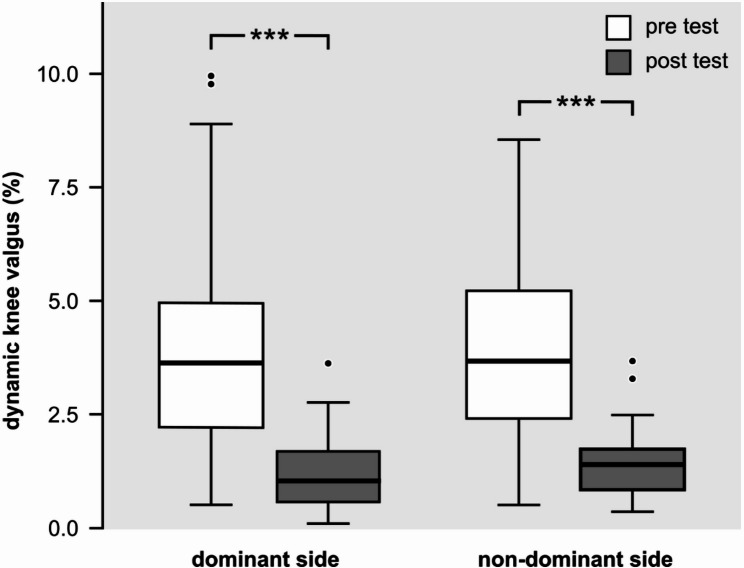
Dynamic knee valgus measured during the single-leg squat test before (white boxes) and after (gray boxes) the training protocol at 15% squat depth. Whiskers represent 1.5 times the interquartile range (IQR), and dots indicate outlier values. *** denotes *p* < 0.001

The extent of the relative dynamic knee valgus during single-leg squat tests reduced significantly after the 6 week-long training program.The participant’s non dominant knee relative latero-medial movement reduced by 2.12%, while this reduction was 2.63% in the dominant knee. The dynamic knee valgus data is shown in the Fig. 1.

## Discussion

The aim of this study was to evaluate the influence of an in-house developed pelvic stabilization training program on dynamic knee valgus. Rationale of this program is based on previous results which pointed out the importance of the interdependency between pelvic muscle force – knee valgus deviation – activity-mediated lower limb injury axis [9, 10, 18].

Evaluation strategy contained both subjective and objective measurement techniques. Our results indicated that there was no change of physical activity before and after the training protocol as reflected from the acquired Tegner scores. Several studies have examined the impact of pelvic and core training on sport activity levels, as measured by the Tegner Activity Scale (TAS), particularly in active individuals and those undergoing rehabilitation. Notably, core stabilization exercises following anterior cruciate ligament (ACL) reconstruction have been shown to significantly improve Tegner scores compared to standard rehabilitation protocols [25]. However, in healthy athletic populations, core training appears to have limited effect on increasing activity levels. A meta-analysis by Prieske et al. (2023) [26] concluded that although core strength programs significantly improve balance and trunk endurance, they do not meaningfully enhance sport-specific performance metrics that would translate into higher Tegner ratings. This findings suggest that while pelvic and core interventions are beneficial in rehabilitation settings, their ability to elevate activity levels in already active individuals is limited unless combined with sport-specific training stimuli [26]. Regarding subjective evaluation of knee status an improvement was recorded by 5.1 points according to pre- and post-test Lysholm questionnaires. Status of general well-being also increased based on the SF-36 battery results. Jung et al. (2022) [27] revealed that incorporating core strengthening into post-injury rehabilitation programs resulted in significant enhancements in knee function, a decrease in pain, and improved joint stability—all of which were reflected in elevated Lysholm scores. Although it is less frequently examined in isolation, training of the hip and core in patients suffering from patellofemoral pain syndrome has also been linked to functional advancements in the aspects measured by the Lysholm scale [28].

Electromyographic evaluation of the trained muscle groups increased without exception. Higher EMG amplitudes indicate the recruitment of larger number of motoric units during muscle contractions. This finding is reflected in the increased observed isometric muscle force after training compared to the pretest values. As expected, our pelvic stabilization training protocol increased core pelvic muscle performance evaluated by both EMG and isometric force measurements. Bilateral stabilization of the pelvis – hip – knee axis resulted significantly attenuated dynamic, horizontal knee displacement throughout the entire squat course. Previous studies have shown that exercises like pelvic tilts, bridges, and side-lying hip abductions enhance gluteal muscle activation as assessed by surface EMG, which suggests improved neuromuscular recruitment [29, 30]. Furthermore, training protocols that focus on the pelvis have been associated with increased maximal voluntary isometric contraction (MVIC) strength of the gluteus medius, thereby contributing to enhanced hip and pelvic control during dynamic movements [31]. The improvement in gluteal strength and activation resulting from pelvic training has been correlated with better lower extremity alignment, a reduction in knee valgus during various activities, and a lower risk of injury [32]. Consequently, pelvic training represents a significant intervention for optimizing gluteal muscle function and improving overall lower limb mechanics. However, despite the widespread endorsement of pelvic training for the enhancement of gluteal muscle EMG activity and strength, certain studies [30, 33] raise concerns regarding the consistency and functional significance of these alterations and they underscore the necessity of incorporating pelvic training into comprehensive, sport-specific programs to facilitate its application to functional performance.

Dynamic knee valgus is determined at 15% of single leg squat depth (relative to lower limb length) on the way down. Based on our earlier findings, knee valgus tendencies are already evident at this level and well tolerable even for pre/postoperative patients with knee problems [34]. Pelvic and core training enhance dynamic knee valgus by improving the strength of hip abductors and external rotators, as well as neuromuscular control, which are essential for stabilizing the pelvis and regulating femoral movement, specific pelvic exercises are effective in restoring appropriate hip and pelvic mechanics, which in turn reduces knee valgus angles and may lower the risk of injury [35, 36]. Improvements in neuromuscular timing and proprioception further enhance knee joint alignment during movement [37]. There is systematic evidence that supports the efficacy of hip and core strengthening interventions in reducing dynamic knee valgus in both healthy individuals and clinical populations [38].

Minimizing dynamic knee valgus, especially at shallow squat depths (approximately 15%), can lower the risk of future lower-extremity injuries. This encompasses acute non-contact ACL tears during dynamic movements, the long-term onset of patellofemoral pain syndrome, and the possible advancement toward osteoarthritis. These results underscore the significance of interventions aimed at enhancing frontal-plane knee control to support joint health and avert injuries. Dynamic knee valgus is a complex functional problem therefore it is clinically important to identify the main causes and the solutions of this compensatory movement and to adapt these findings individually.

As limitations, this study did not include ankle measurements, which could be a limitation of the study, as it is known that ankle deficits could also cause changes in dynamic knee valgus, as well, the sample size and not using a control group could be a limitation to this study. In future investigations we could focus also in ankle corrections, however this study was focusing on pelvic stabilization.

## Conclusions

Based on our results pelvic stabilization training has good benefits on dynamic knee valgus, strength, and muscle activity around the pelvis. Thus, pelvis stabilization training is highly recommended for ACL injured athletes to prevent possible re-traumatization.

## Data Availability

The datasets used during the current study are available from the corresponding author on reasonable request.

## Abbreviations

ACL: Anterior cruciate ligament
DKV: Dynamic knee valgus
MVIC: Maximal voluntary isometric contraction
PFPS: Patello-femoral pain syndrome
TAS: Tegner Activity Scale
